# Calibration Research: Where Do We Go from Here?

**DOI:** 10.3389/fpsyg.2012.00229

**Published:** 2012-07-09

**Authors:** Linda Bol, Douglas J. Hacker

**Affiliations:** ^1^Department of Educational Foundations and Leadership, Old Dominion UniversityNorfolk, VA, USA; ^2^Department of Educational Psychology, University of Utah, Salt Lake CityUT, USA

**Keywords:** calibration, metacognition, self-regulated learning, social cognition, research methods

## Abstract

Research on calibration remains a popular line of inquiry. Calibration is the degree of fit between a person’s judgment of performance and his or her actual performance. Given the continued interest in this topic, the questions posed in this article are fruitful directions to pursue to help address gaps in calibration research. In this article, we have identified six research directions that if productively pursued, could greatly expand our knowledge of calibration. The six research directions are: (a) what are the effects of varying the anchoring mechanisms from which calibration judgments are made, (b) how does calibration accuracy differ as a function of incentives and task authenticity, (c) how do students self-report the basis of their calibration judgments, (d) how do group interactions and social comparisons affect calibration accuracy, (e) what is the relation between absolute and relative accuracy, and (f) to what extent does calibration accuracy predict achievement? To help point the way to where we go from here in calibration research, we provide these research questions, propose research methods designed to address them, and identify prior, related studies that have shown promise in leading the way to fill these gaps in the literature.

Calibration has been defined as the degree of fit between a person’s judgment of performance and his or her actual performance (Keren, 1991). As such, calibration reflects a metacognitive monitoring process that provides information about the status of one’s knowledge or strategies at a cognitive level (Nelson, 1996). Based on this information, control at a metacognitive level can be exerted to regulate one’s knowledge or strategies. Therefore, greater accuracy in a person’s judgments of performance (i.e., being well calibrated) creates greater potential for self-regulation (Zimmerman and Moylan, 2009).

The broad research literature in educational psychology and the more specific literature on self-regulated learning reveal a growing interest in calibration that is well-warranted. For instance, students studying for a test need to be accurate in monitoring their knowledge acquisition and retention if they hope to successfully control further study. On one hand, students may develop a false sense of their mastery of studied material and overestimate how well they will perform. These students’ positive biases could lead to premature termination of study and place them at risk for failure (Hacker et al., 2008a). On the other hand, students may underestimate how well they will perform. These negative biases also can be detrimental to academic performance because students may fail to disengage from studying and misallocate study time if they assume the material is not yet mastered. When students demonstrate strong biases in their calibration judgments, they may not take the remedial steps necessary to improve or evaluate their responses during or after an exam (Hacker et al., 2008b).

Although an exhaustive review of the research on calibration is beyond the scope of this paper, there are some consistent findings. Many studies have indicated that calibration accuracy is linked to achievement level (e.g., Hacker et al., 2000; Grimes, 2002; Bol et al., 2005; Nietfeld et al., 2005). In general, higher-achieving students tend to be more accurate but more underconfident when compared to their lower-achieving counterparts. Another consistent finding is that postdictions are typically more accurate than predictions (Pressley and Ghatala, 1990; Maki and Serra, 1992). This phenomenon makes intuitive sense because a person should be better able to judge how he or she performed after the completion of the task due to familiarity and exposure to the task itself (Hacker et al., 2000). However, task difficulty also influences calibration accuracy. Juslin et al. (2000) identified the *hard-easy effect* in which students tend to be more accurate but underconfident on easy items and less accurate but overconfident on difficult items.

However, other findings have been less consistent and some areas of investigation have not yet been broached. Our purpose is to propose a research agenda that will shed light on the inconsistent findings and address those areas of research that have not yet received attention. We will propose our agenda using Zimmerman and colleagues’ social cognitive model of self-regulation, specifically their personal feedback loop, as a theoretical foundation on which research can be guided (Schunk and Zimmerman, 1997; Zimmerman, 2008; Zimmerman and Moylan, 2009). Briefly stated, self-regulation depends on this personal feedback loop, which provides a person with the necessary information about the status of one’s knowledge or strategies. The self-regulatory feedback consists of three cyclical phases: forethought, performance, and self-reflection (see Figure 1).

**Figure 1:**
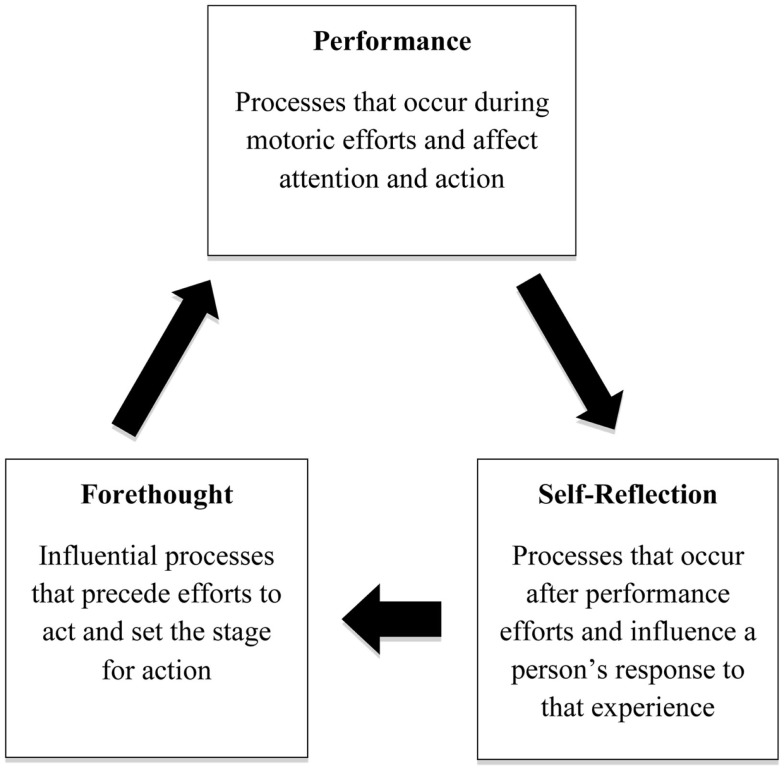
**Zimmerman’s (2000) cyclical model of self-regulation**.

The forethought phase sets the stage for action by providing information about the components of the task at hand, what goals and strategies need to be initiated, and whether the learner has the self-efficacy and self-motivation to accomplish the task. Learners may have a difficult time accurately self-assessing each of these areas of forethought for several reasons, two of which we address here. Estimates of performance have been shown to be biased toward some initial anchor, and learners often do not adequately adjust from these anchors. Therefore, knowing the psychological bases for these anchors and how learners can debias their judgments is not a trivial matter (research question a). In addition, because studies on the effects of incentives on motivation to achieve greater accuracy have been mixed, greater attention needs to be focused on how motivation can be manipulated (research question b).

During the performance phase, the learners gain feedback concerning self-control and self-observation, processes that are essential for continued attention to and action on a task. Learners self-explanations about how and why they use their self-observations to self-control and whether those self-explanations are mediated or moderated by other factors such as attributional style (research question c) or social influences (research question d) are critical areas of investigation. Maintaining attention and action on a task also requires that ongoing performance is being judged accurately. Performance can be judged at a global level (e.g., How prepared is a learner for an upcoming test?) and at local levels (e.g., Is the answer to this question correct?). Knowing whether there is a relation between these global and local levels of judgment can provide insights into the psychological mechanisms upon which they are made (research question e).

Finally, during the self-reflection phase, the learner makes self-judgments and self-reactions on their performance. This feedback on whether actual achievement, the end product of self-regulation, is high or low, satisfactory or unsatisfactory, or simply just good enough, then exerts an influence on whether further action will be taken in the forethought phase. Therefore, knowing whether there is a payoff to self-regulation is instrumental to continued self-regulation (research question f).

## Proposed Directions

Our attention now turns more specifically to our proposed research agenda on how knowledge of calibration can be promoted in further lines of inquiry. Table 1 presents the six research areas and questions already addressed as well as designs and variables aligned with these questions. In addition, we identify prior, related studies that have shown promise in filling these gaps in the literature.

**Table 1:** **Proposed questions, design, and exemplar studies in calibration research**.

Research questions	Design	Variables
What are the effects of varying the anchoring mechanisms from which calibration judgments are made?	True experimental	*Treatment*: manipulating initial judgments *Measures*: extent and type of adjustments for judgments
How does calibration accuracy differ as a function of incentives and task authenticity?	Comparative	*Independent variables*: type of task, and incentive *Measure*: calibration accuracy
How do students self-report the basis for their calibration judgments?	Qualitative	*Measures*: interviews and think-aloud protocols
How do group interactions and social comparisons affect calibration accuracy?	Experimental, factorial	*Treatment*: individual or group settings with or without social comparisons
		*Measures*: calibration accuracy, group interactions
What is the relationship between absolute and relative accuracy?	Correlational	*Measures*: absolute and relative accuracy on items, topics/concepts, and overall performance
To what extent does calibration accuracy predict achievement?	Correlational	*Measures*: calibration accuracy and achievement

### What are the effects of varying the anchoring mechanisms from which calibration judgments are made?

In their seminal article, *Judgment under Uncertainty: Heuristics and Biases*, Tversky and Kahneman (1974) proposed that people make estimates of their performance by starting from some initial value and make adjustments that are biased toward that initial value. Their claim was that these adjustments are often insufficient so that the estimates continue to appear biased. The anchoring-and-adjustment effect has been widely recognized in the decision-making literature (e.g., Mussweiler et al., 2000; Epley and Gilovich, 2004, 2005), and it has been used to explain underconfidence in calibration research (e.g., Scheck et al., 2004).

Researchers of anchoring-and-adjustment effects make a distinction about who sets the initial value from which subsequent adjustments are made. In some cases, the initial value is set by another person (e.g., a salesperson setting the price for a new car) or by oneself (e.g., when guessing how long it takes Mars to orbit the sun, people often select an anchor on Earth’s orbit and then adjust from that value; Epley and Gilovich, 2004). These self-generated anchors are the ones that we believe could potentially influence people’s calibration judgments.

Scheck and Nelson (2005) used anchoring-and-adjustment as an explanation for the underconfidence-with-practice (UWP) effect proposed by Koriat et al. (2002). The UWP effect is a robust finding in which people initially show overconfident calibration when making judgments of learning (JOLs) but subsequently become underconfident after their second study trial. Scheck and Nelson (2005) hypothesized that people form a psychological anchor for their JOLs somewhere between 30 and 50% of correct recall and adjust their JOLs either upwards or downwards based on whether performance is above or below this band. They found that when performance was above 50% after the second study trial, participants adjusted both immediate and delayed JOLs downward in relation to recall, thereby appearing to be underconfident. When performance was below 30% after the second study trial, participants adjusted their delayed JOLs upwards in relation to recall, thereby appearing to be overconfident; and when performance was at 30%, their immediate JOLs were near perfect. If accuracy of calibration is a necessary condition for self-regulated learning, then a potent research question is how can higher achievers overcome their underconfident JOLs and lower achievers overcome their overconfident JOLs without adversely impacting students who are between the two groups?

Research into this question can be informed by research conducted by Epley and Gilovich (2005) in which insufficient adjustments were compensated by providing financial incentives that motivated participants to make additional adjustments. Also, forewarning participants that initial adjustments are inadequate helped them to engage in additional adjustments, resulting in greater accuracy. Finally, Zhao and Linderholm (2011) found that providing information about peer performance on a task can serve as an anchor for metacomprehension judgments and can be used to debias judgments. We propose initiating research on this topic by posing the following question, “What are the effects of varying the anchoring mechanism from which calibration judgments are made?” Experimental work following any one of these lines of research could be pursued to demonstrate viable ways to encourage adjustment away from initial judgments and toward greater calibration accuracy.

### How does calibration accuracy differ as a function of incentives and task authenticity?

Granted, motivation is a broad construct, but as in other areas of education research, calibration cannot be thoroughly understood without reference to motivational variables. To narrow the focus of this line of inquiry, we have selected incentives and task authenticity as starting points. These represent reasonable starting points because they represent salient constructs in the forethought phase of self-regulated learning and can be readily generalized to classroom contexts. In our conceptualization, incentives would be some type of course credit, and task authenticity would be learning course content. Focusing on learning course content stands in stark contrast to typically used tasks in calibration research, such as learning paired associates in a remote tribal language to control for prior learning.

Studies examining the impact of incentives on calibration are rare. In the preceding section, we referenced Epley and Gilovich’s (2005) study in which financial incentives were employed to motivate participants to adjust their metacognitive judgments. In another more ecologically valid study, we manipulated incentives and reflection in a fully crossed quasi-experiment conducted in college course (Hacker et al., 2008a). Students in an extrinsic reward condition were told they would receive one to four additional points on each of three exams, depending on their calibration accuracy, with more points given for greater accuracy. We found that incentives significantly improved calibration accuracy but only among lower-achieving students, which may have been the result of greater motivation on their part to be accurate to earn the additional points. Schraw et al. (1993) also provided evidence for the effectiveness of incentives for promoting calibration accuracy. In their procedure, students also received extra credit either for improving performance on a test or increasing their calibration accuracy. Though performance improved in both incentive conditions, the findings revealed that incentivizing accurate calibration was more effective than incentivizing improved performance.

Thus, the research question we propose is how does calibration accuracy differ as a function of incentive or task authenticity? Because it would be difficult to isolate these variables while controlling for all other influences, comparative studies might be more appropriate initially. In future research, the influence of both incentives and task authenticity could be investigated in the same study. For example, in a within-subjects design, calibration accuracy might be compared for students completing more authentic versus contrived tasks in conditions where incentives are or are not present.

### How do students self-report the basis for their calibration judgments?

Earlier we posed a research question related to how individuals might anchor and then adjust their calibration. What we have not adequately addressed is how students self-report the basis for calibration judgments. Gathering self-report data from students regarding their calibration judgments is rare but not unprecedented. In a mixed methods study, Hacker et al. (2008a) asked college students to identify factors that influenced the accuracy of their predictions and postdictions. Attributional style was used as the theoretical framework to organize the data. The most frequent explanations focused on internal, student-centered constructs. Students were most likely to attribute discrepancies between their scores and calibration judgments to how much or how well they studied or how well they felt they knew the material. Another frequently reported factor was test-taking ability and prior performance on tests as well as expectations for test content and difficulty. Similar results were reported in a study with middle school students (Bol et al., 2010). In this study, immediately after making their predictions, students were asked why they predicted that score. The most frequent categories of responses centered on time and effort spent studying, global perceptions of their own abilities, and past performance. After making their postdictions, students again were asked to explain why these were accurate or inaccurate. Explanations focused on knowing the number answered correctly, their expectations of test difficulty, the effort exerted in studying, and their global sense of self-confidence.

Bandura’s model of reciprocal determinism has also been used to categorize responses (Dinsmore and Parkinson, 2012). Participants were asked to explain how they arrived at or what was considered when making confidence judgments. Instances of the *a priori* categories observed in student responses included prior knowledge, characteristics of the text and the items, and guessing. Students often cited a combination of personal and task characteristics. The combination of how students explain their calibration judgments and how judgments are measured may be important for understanding how judgments contribute to performance.

Although the results from a few studies have helped us understand how students describe the basis for their calibration judgments, more research is warranted. As employed in the studies just described, a qualitative approach grounded in a theoretical framework would be most appropriate and revealing. Qualitative data could be collected via open-ended responses to surveys and think-aloud protocols in which real-time data would be collected as students are considering, making, and explaining their calibration judgments.

Threats to validity in self-reported data cannot be avoided. Consequently, in the qualitative tradition, we might rely on triangulation strategies to support the credibility or transferability of findings. Researchers (Azevedo et al., 2010; Winne, 2010) recommend combining self-report data about self-regulated learning with trace evidence that reflects students’ cognitive operations (e.g., highlighting text). Data collected in computer-based learning environments should facilitate these types of studies. For example, if students attribute their lack of understanding of a topic to lack of study time, actual time spent studying the topic could be calculated. We might also follow their navigation patterns to determine whether they return to content judged to be in need of further study.

### How do group interactions and social comparisons affect calibration accuracy?

Experimental manipulations centered on group interactions and social comparisons have already shown promise in improving calibration accuracy. In our recent factorial experiment with high school biology students (Bol et al., 2012), half of the students practiced calibration in groups while the other half practiced calibration individually. The second treatment variable was whether students used guidelines to gage their judgments of how well they mastered the content. We found both group settings and guidelines to be effective in promoting calibration accuracy and achievement. Other studies have demonstrated that the combination of group learning contexts and guiding questions promoted metacognitive skills and achievement (Kramarski and Mevarech, 2003; Kramarski and Dudai, 2009).

Group work logically elicits implicit or explicit social comparisons. Carvalho and Yuzawa (2001) manipulated social comparisons by presenting some participants with information concerning the mean percentage of correctly answered questions that a fictitious group of fellow students presumably scored. This information was presented prior to participants making their own metacognitive judgments on their performance. The results indicated that social comparisons did impact the magnitude of metacognitive judgments with greater magnitude in judgments associated with higher performance. Other results suggested that participants with little confidence in their judgments may be particularly susceptible to social influences.

Given that group settings and social comparisons can influence metacognitive judgments, the next logical step would be to manipulate both of these variables in a factorial experiment. The question posed is how do group interactions and social comparisons affect calibration accuracy? Students would be asked to calibrate in group or individual settings and would do so with or without social comparisons. Because earlier studies suggest that guidelines promote accuracy in metacognitive judgments (Kramarski and Dudai, 2009; Bol et al., 2012), all four groups would receive guidelines. The social comparisons could be presented to half of the students as part of the guidelines employed by students, and could take the form of mean accuracy scores achieved by low, middle, and high achievers. However, rather than using fictitious scores as in the Carvalho and Yuzawa (2001) research, actual scores could be presented, which may help students differentiate between more and less reasonable calibration judgments and how they may be tied to achievement levels. This may be especially beneficial for lower-achieving students who tend to overestimate their performance (e.g., Bol et al., 2010). Noting the group interactions among students assigned to this condition may further illuminate how social interactions or comparisons may affect calibration judgments.

### What is the relationship between absolute and relative accuracy?

Calibration (aka absolute accuracy) provides estimates of overall memory retrieval (Lichtenstein et al., 1982; Keren, 1991; Nietfeld et al., 2006), and relative accuracy (aka discrimination) provides estimates of whether a person’s judgments can predict the likelihood of correct performance of one item relative to another (Nelson, 1996). For instance, a student can judge that overall he or she will get 85% of the items on a test correct and in fact get 85% correct (i.e., perfect calibration accuracy), but upon closer examination, the student may have given high confidence judgments to items answered incorrectly and low confidence to items answered correctly, in which case, relative accuracy may be close to chance. Both types of accuracy are important for students to self-regulate their learning; however, whether there are shared psychological processes contributing to both is an important area for investigation (Maki et al., 2005).

Accuracy of both global and item-level calibration judgments plays an important role in current and future study efforts because poor judgments can lead to either premature or protracted termination of study of general and specific content. Current research of metacomprehension judgments has shown that there is little or no relation between absolute and relative accuracy (Maki et al., 2005). However, other areas of metacognitive research have not received much attention. For example, we are not aware of any research that has examined absolute and relative accuracy for a typical classroom exam, consisting of multiple-choice or true/false items. If there is a relation between the two, students’ overall global judgments about what they know about the to be tested material may be based on an appraisal of knowing specific and well-defined concepts from that material. In that case, the psychological processes that contribute to one may help to inform the other. However, if there is no relation between the two, then either there is a mismatch between what students believe is to be tested and what is actually tested, or the global judgments may not be helpful because they provide no indication of how students will perform on specific test knowledge.

Therefore, future correlational research could examine whether there a relation between absolute accuracy at the test-level and relative accuracy at the item-level. If such a relation exists, whether it varies by item difficulty or type would be important to examine further. Moreover, if this relation exists, developing interventions that could capitalize on it and provide students with ways of better judging item-level and test-level knowledge to prepare for tests would be important contributions to self-regulated learning.

### To what extent does calibration accuracy predict achievement?

Although widespread acceptance has been given to the theoretical argument that accurate metacognitive monitoring is essential to self-regulated learning, the empirical question of whether achievement is enhanced because of accurate monitoring has received surprisingly scant attention. Correlational and experimental studies have established that monitoring can positively impact decisions about what to study (e.g., Metcalfe and Finn, 2008; Hines et al., 2009); but whether that studying leads to gains in achievement is a question in need of further support (Dunlosky and Rawson, 2011). Some calibration studies have shown a positive relation between calibration accuracy and achievement level (e.g., Hacker et al., 2000; Bol and Hacker, 2001). In addition, Nietfeld et al. (2006) and Bol et al. (2012) demonstrated that students who participated in interventions to increase calibration accuracy realized higher gains in achievement than students who did not participate in them. However, in both these studies, treatment and classroom assignment were conflated, leaving open the question of whether internal validity was potentially compromised.

Dunlosky and Rawson (2011) experimentally manipulated judgment accuracy by asking participants to study key-term definitions, one group used an idea-unit standard in which the participants were shown their responses with the idea units contained in the correct answer, and another group used their responses but without access to the correct answer. After being shown their responses, participants made a self-score judgment about whether their answer was correct. The test-judge-study trails continued until a definition was judged as correct three times. Two days later, all participants were administered a retention test. Findings indicated that greater accuracy was related to greater retention. Moreover, they found that participants who were overconfident in their judgments prematurely terminated study, and as a consequence their retention suffered.

Similar experimental manipulations of accuracy need to be conducted to more firmly establish the link between calibration accuracy and achievement. Empirical findings provide multiple ways to manipulate accuracy: study guidelines used in group settings (Bol et al., 2012), self-assessment with feedback (Nietfeld et al., 2006), and feedback with idea-unit standards (Dunlosky and Rawson, 2011). Employing these accuracy manipulations and measuring subsequent retention could provide valuable support for the importance of accurate monitoring and provide information about whether specific manipulations of accuracy lead to greater retention. In addition, the kinds of learning could be manipulated. The tasks used in an experiment could be varied from simple paired associates to multiple-choice tests to text comprehension. Firmly establishing the link between monitoring accuracy and achievement is a critical goal for calibration research.

## Summary and Conclusion

We do not assume that these are the only fruitful research directions to pursue in order to more thoroughly understand calibration and its role in promoting self-regulated learning. However, they represent a good start. The research agenda outlined is based on the social cognitive model of self-regulation developed by Zimmerman and his colleagues (Schunk and Zimmerman, 1997; Zimmerman, 2008; Zimmerman and Moylan, 2009). In alignment with the forethought phase we propose investigating the effects of anchoring, incentives, and task authenticity on calibration judgments to reflect psychological processes linked to self-efficacy and motivation. Research questions focused on social influences, self-explanations, and the basis for metacognitive judgments are represented in the performance phase of the model. More specifically, we propose addressing student explanations for calibration judgments, the impact of group interactions and social comparison on calibration accuracy, and the relationship between absolute and relative accuracy. In the self-reflection phase, learners judge and react to their performance or achievement. The final question reflects the extent to which calibration accuracy predicts achievement. Feedback on performance and self-reflection influences subsequent, cyclical phases of self-regulated learning.

Similarly, the methods we propose are not exhaustive and reflect examples of how these questions may be pursued. The methods we propose range from qualitative approaches investigating how students explain their calibrations judgments, comparing motivational factors linked to task characteristics, correlating absolute and relative accuracy, predicting achievement based on calibration accuracy, and manipulating anchoring and adjustment effects as well as social interactions in controlled experiments.

Calibration research will be further advanced when we identify patterns of findings guided by sound theoretical models and based on precise descriptions of terms, measures, contexts, tasks, and populations. As we have argued previously (Hacker et al., 2008b), calibration has been measured in different ways but largely studied in more contrived contexts using college students. Granted, we must consider the trade-off between internal and external validity as we move into more naturalistic settings, such as classrooms and employ more authentic tasks. Various research methods with varying levels of control will better inform our questions overall.

## Conflict of Interest Statement

The authors declare that the research was conducted in the absence of any commercial or financial relationships that could be construed as a potential conflict of interest.
